# Anti-Diabetic Efficacy and Impact on Amino Acid Metabolism of GRA1, a Novel Small-Molecule Glucagon Receptor Antagonist

**DOI:** 10.1371/journal.pone.0049572

**Published:** 2012-11-19

**Authors:** James Mu, Sajjad A. Qureshi, Edward J. Brady, Eric S. Muise, Mari Rios Candelore, Guoqiang Jiang, Zhihua Li, Margaret S. Wu, Xiaodong Yang, Qing Dallas-Yang, Corey Miller, Yusheng Xiong, Ronald B. Langdon, Emma R. Parmee, Bei B. Zhang

**Affiliations:** Discovery and Preclinical Sciences, Merck Research Laboratories, Merck Sharp & Dohme Corp., Whitehouse Station, New Jersey, United States of America; Broad Institute of Harvard and MIT, United States of America

## Abstract

Hyperglucagonemia is implicated in the pathophysiology of hyperglycemia. Antagonism of the glucagon receptor (GCGR) thus represents a potential approach to diabetes treatment. Herein we report the characterization of GRA1, a novel small-molecule GCGR antagonist that blocks glucagon binding to the human GCGR (hGCGR) and antagonizes glucagon-induced intracellular accumulation of cAMP with nanomolar potency. GRA1 inhibited glycogenolysis dose-dependently in primary human hepatocytes and in perfused liver from hGCGR mice, a transgenic line of mouse that expresses the hGCGR instead of the murine GCGR. When administered orally to hGCGR mice and rhesus monkeys, GRA1 blocked hyperglycemic responses to exogenous glucagon. In several murine models of diabetes, acute and chronic dosing with GRA1 significantly reduced blood glucose concentrations and moderately increased plasma glucagon and glucagon-like peptide-1. Combination of GRA1 with a dipeptidyl peptidase-4 inhibitor had an additive antihyperglycemic effect in diabetic mice. Hepatic gene-expression profiling in monkeys treated with GRA1 revealed down-regulation of numerous genes involved in amino acid catabolism, an effect that was paralleled by increased amino acid levels in the circulation. In summary, GRA1 is a potent glucagon receptor antagonist with strong antihyperglycemic efficacy in preclinical models and prominent effects on hepatic gene-expression related to amino acid metabolism.

## Introduction

Glucagon is a 29 amino acid polypeptide hormone that is secreted by pancreatic alpha cells primarily during the fasting state [1]. It plays a critical role in glucose homeostasis and the prevention of hypoglycemia, primarily by promoting glycogenolysis and gluconeogenesis in the liver and attenuating inhibition of these processes by insulin [2], [3]. Hyperglucagonemia has been associated with hyperglycemia in diabetic humans and animal models [3]–[5] and may play an important role in hyperglycemia that is associated with insulin deficiency [3], [6]. There has thus been considerable interest in the development of therapeutic interventions that would ameliorate hyperglycemia by reducing circulating levels of glucagon or inhibiting glucagon actions in target tissues [7]–[9].

The action of glucagon on target organs is mediated via the glucagon receptor (GCGR), a member of the family B seven transmembrane G-protein coupled receptor superfamily found primarily in the liver [2], [3], [10]. Glucagon binding to the GCGR leads to activation of adenylyl cyclase and the biological effects of glucagon are mediated primarily through increased intracellular levels of cAMP [3], [9], [10]. In the mouse, targeted disruption of the GCGR gene results in reduced plasma glucose concentrations [11], [12] and treatment with GCGR antisense oligonucleotides has an antihyperglycemic effect in rodent models of diabetes [13], [14]. Neither approach to disruption of GCGR function results in overt hypoglycemia; this suggests that pharmacotherapy aimed at antagonizing glucagon action at the GCGR may provide useful reductions in blood glucose without significantly increasing risk for hypoglycemia. The phenotype of GCGR knockout mice does, however, include some potentially troublesome features; GCGR mice have prominent α-cell hyperplasia and very high plasma concentrations of glucagon and both active and inactive GLP-1 [12], [15].

A number of small-molecule GCGR antagonists (GRAs) have been developed and have demonstrated, in studies done in preclinical species, prominent antihyperglycemic efficacy that is sustained during chronic dosing. In addition, they have been shown to attenuate blood glucose excursions that are induced by exogenous glucagon and to increase blood levels of the incretin glucagon-like peptide-1 (GLP-1) [16]–[21]. As concerns the potential for untoward actions, it has been reported that chronic GRA treatment of mice does not produce hyperplasia of alpha cells or very large increases in plasma glucagon or GLP-1 [19], [20]. Glucagon-induced gluconeogenesis involves hepatic catabolism of glucogenic amino acids [22]–[24], and knockout of the GCGR gene has been shown to have prominent effects on liver and plasma amino acids in mouse [24], [25]. However, potential effects of GRAs on amino acid metabolism have not been studied.

Here, we report findings from preclinical studies of GRA1, a novel GRA, demonstrating its potential utility for the treatment of hyperglycemia. The present data include characterization of GRA1's substantial antihyperglycemic efficacy in 3 rodent models of diabetes, various findings relating to its potential safety and tolerability, an analysis in the monkey of GRA1 treatment effects on hepatic gene expression related to amino acid metabolism, and GRA1 effects on plasma concentrations of glucogenic amino acids in the monkey.

## Materials and Methods

### Ethics Statement

All animal procedures were reviewed and approved by the Institutional Animal Care and Use Committee of Merck & Co., Inc.

### Materials

**Figure 1:**
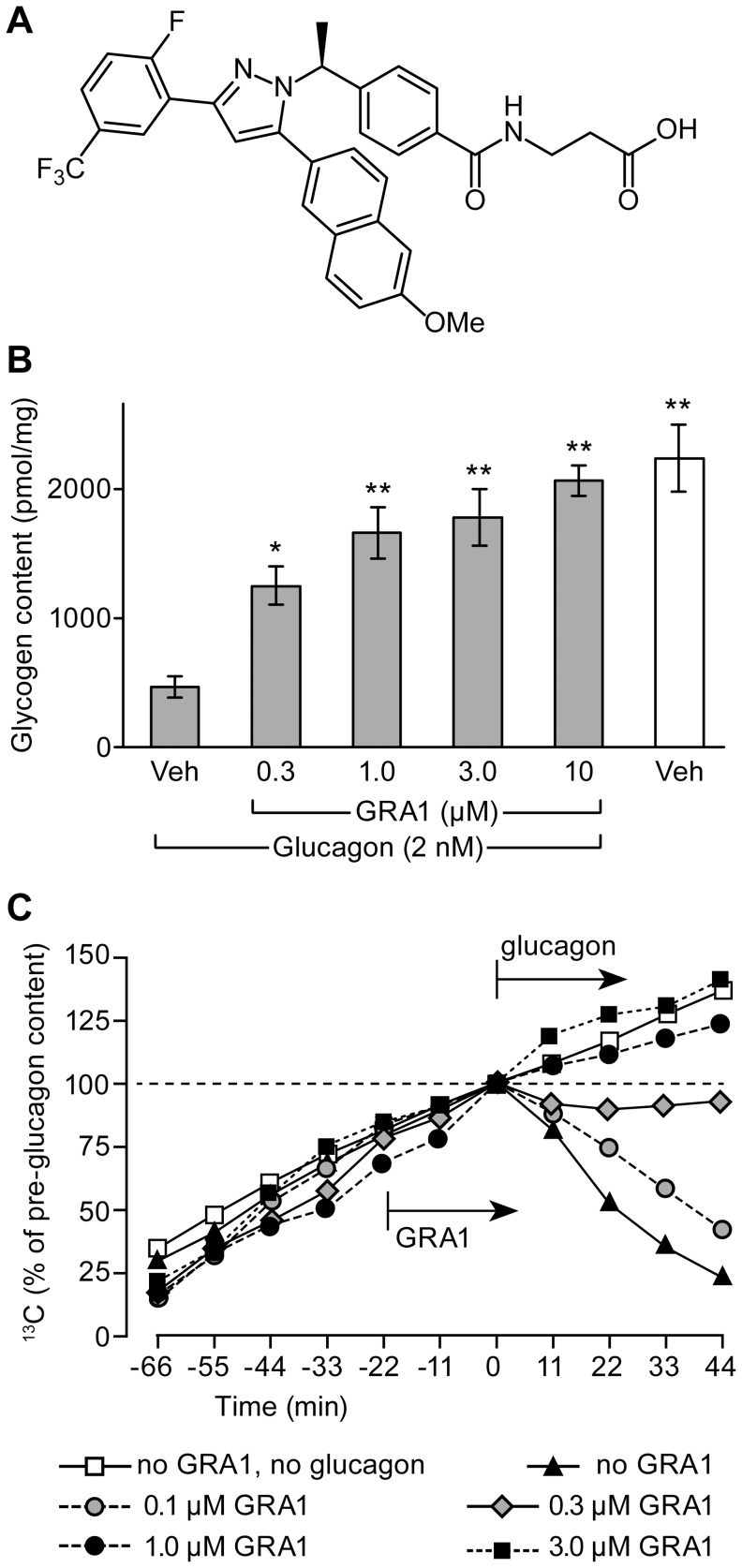
GRA1 inhibits glucagon-mediated glycogenolysis *in vitro* and *ex-vivo*. (A) The chemical structure of GRA1. (B) Dose-dependent inhibition of glucagon-stimulated glycogenolysis in human hepatocytes. The cells were pretreated for 3 h with 200 nM insulin and then challenged with 2 nM glucagon with vehicle (Veh, medium with 1% DMSO) or 0.3–10 µM GRA1. Glycogen content was measured after incubating for 1 h. *p<0.05 and **p<0.01 vs. glucagon+vehicle alone. (C) Inhibition of glucagon-stimulated glycogenolysis in perfused liver prepared from the hGCGR mouse. Livers were perfused with Ringers solution containing 6.7 mM [2-^13^C]pyruvate while ^13^C content (indicative of accumulated [^13^C]glycogen) was monitored dynamically by nuclear magnetic resonance imaging. At time = −22 min, 0.1–3.0 uM GRA1 or vehicle was added to the perfusate; 50 pM glucagon or vehicle was added at time 0. The data are means ±SEM.

All chemicals and reagents were procured from commercial sources except for GRA1 (*N*-[4-((1*S*)-1-{3-(2-fluoro-5-trifluoromethylphenyl)-5-[6-methoxynaphth-2-yl]-1*H*-pyrazol-1-yl}ethyl)benzoyl]-β-alanine, Fig. 1A) [26] and des-fluoro-sitagliptin (7-[(3*R*)-3-amino-1-oxo-4-(2,5-difluorophenyl)butyl]-5,6,7,8-tetrahydro-3-(trifluoromethyl)-1,2,4-triazolo[4,3-*α*]pyrazine); these were synthesized in-house.

### *In Vitro* Assays

Transfected Chinese hamster ovary (CHO) cell lines were acquired and maintained as previously described [17], [20]. These included separate cell lines stably expressing human GCGR (hGCGR), mouse GCGR, rhesus GGCR, glucose-dependent insulinotropic peptide receptor (GIPR), GLP-1 receptor (GLP-1R), pituitary adenylate cyclase-activating polypeptide receptor type 1 (PAC1R), and vasoactive adenylate cyclase-activating polypeptide receptor type 2 (VPAC2R). Inhibition of glucagon binding to hGCGR was assayed in cell membranes prepared from the line of CHO cells that expressed the hGCGR. Functional antagonism was assayed by measuring the production of cAMP in intact CHO cells stimulated by agonists specific for their cognate receptors (i.e., glucagon for GCGR-expressing cell lines, GIP for the GIPR-expressing line, etc.). Primary human hepatocytes were obtained from In Vitro Technologies (Baltimore, MD) and glucagon-stimulated glycogenolysis was studied in these as previously described [17].

### Animals

The present study made use of a line of hGCGR-expressing mouse (“hGCGR mouse”) generated previously by substituting the hGCGR gene for the mouse GCGR gene [27]. These mice were crossed with C57BL/6J.*ob/+* mice (Jackson Laboratories, Bar Harbor, ME) [28] to obtain an hGCGR.*ob/+* mouse line. The hGCGR.*ob/+* mice were then intercrossed to obtain hGCGR.*ob/ob*, hGCGR.*ob/+*, and hGCGR.*+/+* mice (with the hGCGR.*ob/+* and hGCGR.*+/+* mice serving as littermate controls). Diabetes-induced obese (DIO) hGCGR mice were generated by maintaining hGCGR mice on a high-fat diet (HFD) in which 45–60% of calories were in fat (Product S3282, Bio-Serv, Frenchtown, NJ) [20]. High-fat-diet-streptozotocin-treated (HFD/STZ) diabetic mice were generated in-house as previously described [15].

The mice were maintained under controlled conditions of lighting (12-h light/dark), temperature (23±2°C), and humidity (55±15%) with access *ad libitum* to mouse/rat diet (7012 Teklad LM-485; Harlan Laboratories, Indianapolis, IN) and water, except as noted. The analyses of plasma amino acid concentrations and hepatic gene expression were done in chair-trained, normoglycemic rhesus monkeys.

### Inhibition of ^125^I-glucagon binding to hGCGR *In Vivo* and *Ex Vivo* Assay of Liver Glycogen Content

Inhibition of ^125^I-glucagon binding to hGCGR (a measure of hGCGR occupancy by antagonist) was assayed in hGCGR mouse liver *in vivo* as previously described [29]. Glycogen content of perfused hGCGR mouse liver was measured as previously described [30]. In brief, livers were perfused *ex vivo* with Ringers solution containing 6.7 mM [2-^13^C]pyruvate while ^13^C content (indicative of accumulated [^13^C]glycogen) was monitored dynamically by nuclear magnetic resonance imaging.

### Attenuation of Blood Glucose Excursions Induced by Glucagon

In mouse studies of glucagon-induced excursions in blood glucose, animals were dosed orally with vehicle (10 mL/kg 0.5% aqueous methylcellulose) or GRA1 one hour prior to challenge by intraperitoneal injection of 15 µg/kg glucagon (Eli Lilly, Indianapolis, IN) or vehicle [17], [20]. In the studies in monkey, GRA1 or vehicle was administered to chair-restrained animals via a nasogastric tube 4 h prior to intramuscular injection of 15 µg/kg glucagon. Blood glucose measurements were made using a OneTouch glucometer (Lifescan, Milpitas, CA).

### Evaluations of Antihyperglycemic Efficacy in the hGCGR.*ob/ob* and HFD/STZ Mouse

Acute glucose lowering was studied in non-fasted hGCGR.*ob/ob* mice and HFD/STZ mice administered single doses p.o. of 1, 3 and 10 mg/kg GRA1 in 10 mL/kg 0.5% aqueous methylcellulose. Animals were fasted during the interval between dosing and the final blood glucose measurement. Glucose was measured in blood from tail bleeds using a OneTouch glucometer. In the experiments with hGCGR HFD/STZ mice, treatment groups were matched with respect to body weight and blood glucose levels.

Animals were dosed chronically with GRA1 and the DPP-4 inhibitor des-fluoro-sitagliptin by administering these agents as food admixtures. The admixtures were prepared in-house and by Research Diets (New Brunswick, NJ), with drug concentrations adjusted weekly on the basis of average food consumption and animal weight to provide targeted daily doses. In the GRA1 experiments, the targeted doses were 3, 6, 10, and 30 mg/kg•day. In des-fluoro-sitagliptin experiments, the targeted dose was 200 mg/kg•day, a relatively high amount necessary because this compound has a half-life in rodents of 1–2 h [31].

### Plasma Hormone Measurements

Plasma glucagon and active GLP-1 were measured using commercial ELISA kits (Linco Research Immunoassay, St. Charles, MO). Plasma inactive GLP-1 was measured using a 96-well ELISA developed in-house [20]. Plasma insulin was determined by ELISA with a commercial kit (ALPCO Diagnostics, Windham, NH). Plasma concentrations of glycated hemoglobin A_1c_ (HbA1c) were measured using a Micromat II test kit from Bio-Rad Laboratories (Hercules, CA) [20]. Total cholesterol, low-density lipoprotein cholesterol (LDL-c) and non-LDL-c, and aspartate aminotransferase were measured using a Roche P Modular Clinical Chemistry analyzer (Indianapolis, IN).

### Evaluation of Hepatic Gene-Expression Levels and Plasma Amino Acid Concentrations in Rhesus Monkey

Hepatic gene expression was assessed by liver biopsy in adult rhesus monkeys that had been treated twice daily for 1 and 6 days with 30 mg/kg GRA1 or vehicle. The compound was administered to the animals orally in yogurt or a similar treat while they were housed in their home cages. The animals were fasted for approximately 16 h prior to performing liver biopsies laparoscopically, under anesthesia. On biopsy days, animals received their morning doses of GRA1 2 h before the biopsies were performed.

Total RNA was processed for Affymetrix microarray analysis as previously described [32]. Briefly, total RNA was isolated from frozen tissues after homogenizing in TRIzol reagent (Invitrogen, Carlsbad, CA) and processed using RNeasy kits (QIAGEN, Valencia, CA) according to manufacturers' instructions. Sample amplification, labeling, and microarray processing were performed by the Covance Genomics Laboratory in Seattle, WA using the Affymetrix catalog Rhesus array (Affymetrix, Santa Clara, CA). One-way ANOVA analyses were performed with Matlab (The Mathworks, Natick, MA). Probesets had to pass a pre-filter of Affymetrix MAS5 present call p value <0.05 in >50% of the samples to qualify for further analysis. Differentially expressed genes (probesets) were selected with 1.2-fold change and ANOVA p value <0.05.

In a follow-up experiment, adult rhesus monkeys were dosed once daily for 14 days with 30 mg/kg GRA1 or vehicle (0.5% methylcellulose plus 0.02% sodium dodecyl sulfate in 2 mL/kg water) delivered by oral gavage. Blood samples were collected on days 0, 1, 7, and 14 after the animals had fasted overnight. Plasma concentrations of glucose, glucagon, and amino acids were assayed by the Hormone Assay & Analytical Services Core at Vanderbilt University (Nashville, TN).

### Statistical Analysis

Data analysis was performed with the aid of GraphPad Prism® software (GraphPad Software, San Diego, CA). Calculations of p-value were based in analysis of variance (ANOVA) and the unpaired student's *t* test, whichever was applicable. Statistical significance was defined as p<0.05.

## Results

### *In vitro* evidence that GRA1 is a potent and selective glucagon receptor antagonist

Binding of ^125^I-glucagon to hGCGR *in vitro* was strongly inhibited by low-nanomolar concentrations of GRA1; the IC_50_ for this inhibition was 4 nM (Table 1). Production of cAMP by glucagon-stimulated hGCGR-expressing CHO cells was inhibited by GRA1 with an IC_50_ of 12 nM. The potency of GRA1 against glucagon-stimulated cAMP production in CHO cells expressing rhesus GCGR was similar to that observed in cells expression hGCGR. However, its potency against glucagon-stimulated cAMP production in cells expressing mouse GCGR was 30-fold lower. Given this potency difference, all subsequent studies in mice were done in a transgenic line that expressed hGCGR instead of murine GCGR [27].

**Table 1:** Potency and specificity of GRA1 *in vitro*.

Receptor	Species	Assay^a^	IC50 (nM)
GCGR	human	binding	4
GCGR	human	cAMP	12
GCGR	rhesus	cAMP	15
GCGR	mouse	cAMP	178
GIPR	human	cAMP	933
GLP-1R	human	cAMP	7900
PAC1R	human	cAMP	7850
VPAC2R	human	cAMP	4200

GCGR = glucagon receptor.

GIPR = glucose-dependent insulinotropic peptide receptor.

GLP-1R = GLP-1 receptor.

PAC1R = pituitary adenylate cyclase-activating polypeptide receptor type 1.

VPAC2R = vasoactive adenylate cyclase-activating polypeptide receptor type 2.

a Inhibition of binding between ^125^I-glucagon and the hGCGR was measured in membranes prepared from CHO cells expressing hGCGR. Inhibition of cAMP production was measured in intact CHO cells stably expressing human GCGR, GIPR, GLP-1R, PAC1R, and VPAC2R and stimulated by their respective agonists, glucagon, GIP, GLP-1, PAC1, and VPAC2.

GRA1 was substantially less potent as an inhibitor of hormone-stimulated cAMP production in cells expressing other family B homologs of GCGR (Table 1). In these cell lines, the observed IC_50_ values for GRA1 inhibition of cAMP production were 78–350-fold higher than in hGCGR-expressing cells stimulated by glucagon.

Glucagon-mediated glycogenolysis in human hepatocytes was profoundly inhibited by GRA1. When not treated with GRA1, glycogen-loaded human hepatocytes rapidly lost approximately 80% of their glycogen following challenge by 2 nM glucagon (Fig. 1B). This response to glucagon was inhibited by approximately 50% in the presence 0.3 µM GRA1 and it was completely prevented by 10 µM GRA1.

Net accumulation of ^13^C in [2-^13^C]pyruvate-perfused liver was rapidly reversed by 50 pM glucagon to the perfusate (Fig. 1C). This hepatic response to glucagon was completely prevented by pretreatment of liver with 1 or 3 µM GRA1. Pretreatment with 0.3 µM GRA1 appeared to reduce the response to glucagon by about 50%.

### Inhibition of ^125^I-Glucagon Binding in the hGCGR Mouse *In Vivo* and Blunting of Glucagon-Induced Excursions in Blood Glucose in hGCGR Mouse and Rhesus Monkey

In hGCGR mice given a single oral dose of 2 mg/kg GRA1, the mean rate of absorption from the gut was 69%, the apparent plasma terminal half-was 6.8 h, and nearly all absorbed drug was subsequently excreted unchanged via the bile. Under control conditions, hGCGR mice were normoglycemic and treatment of these animals with GRA1 lowered blood glucose concentrations only moderately.

**Figure 2:**
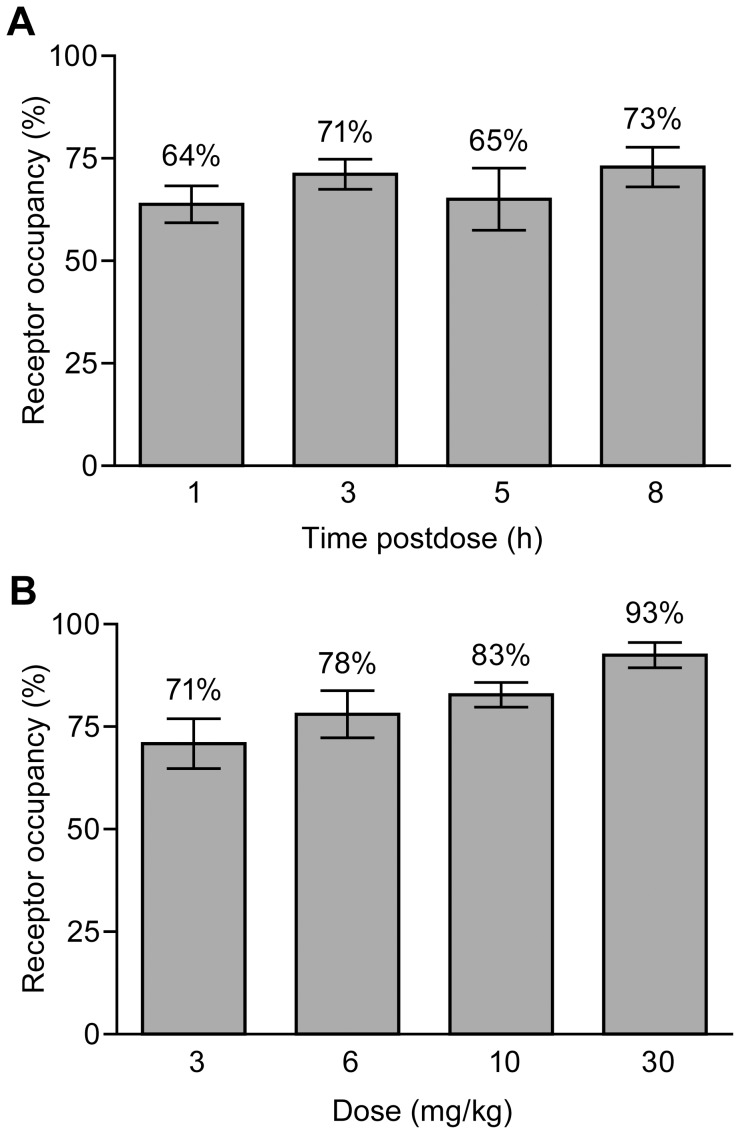
*In vivo* inhibition of hepatic ^125^I-glucagon binding in the hGCGR mouse following (A) acute and (B) chronic dosing with GRA1. The data are mean (±SEM) percent reductions in liver ^125^I-glucagon content measured (A) 1, 3, 5, and 8 h after a single oral dose of 3 mg/kg GRA1, and (B) after treatment for 30 days with control diet or food/drug admixtures that provided 3, 6, 10, or 30 mg/kg·day GRA1. Pharmacokinetic analysis performed during the experiment in (A) determined that mean plasma GRA1 concentrations were 0.5, 0.6, 0.5, and 0.7 µM at 1, 3, 5, and 8 h postdose, respectively.

**Figure 3:**
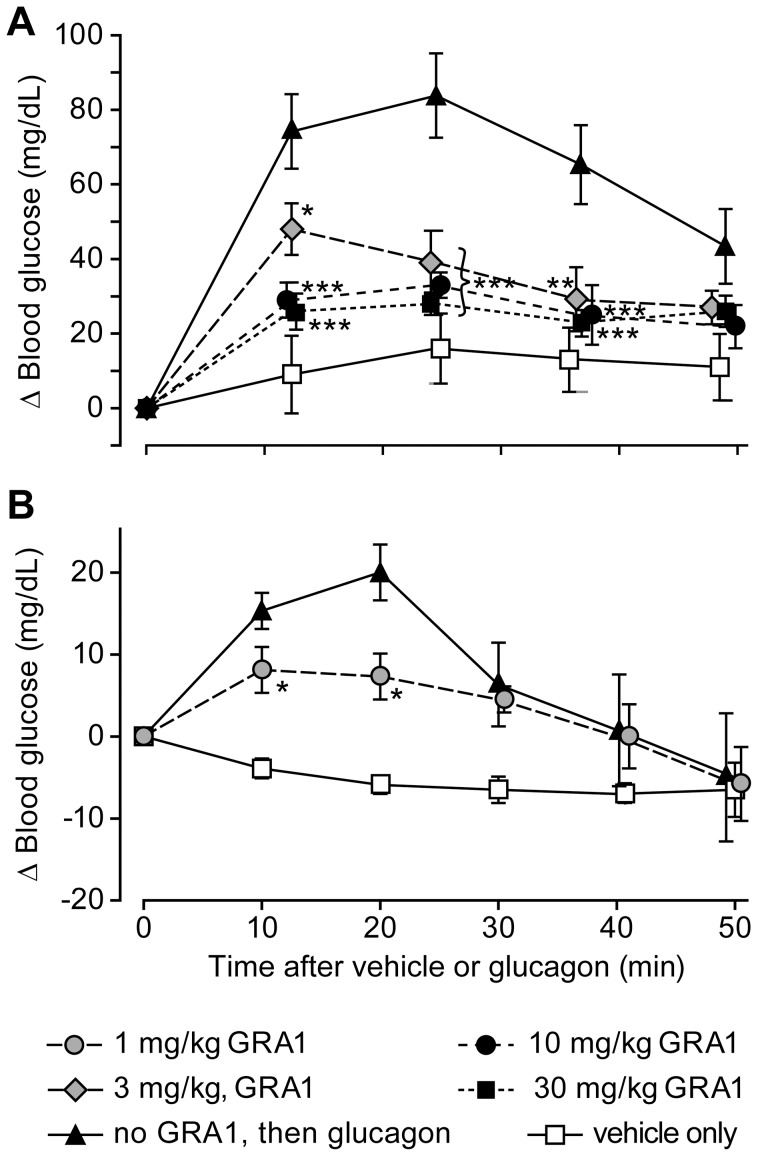
Blunting of glucagon-induced blood glucose excursions by GRA1 treatment in the (A) hGCGR mouse and (B) rhesus monkey. In (A), mice (n = 8) were administered vehicle or 3, 10 or 30 mg/kg GRA1, then challenged 1 h later (time 0) by intraperitoneal injection of 15 µg/kg glucagon or vehicle. Pharmacokinetic analysis determined that these animals had mean plasma GRA1 concentrations of 0.4, 0.9, and 4.7 µM, respectively, at 1 h postdose. In (B), monkeys (n = 4) were administered 1 mg/kg GRA1 4 h prior to intramuscular injection of 15 µg/kg glucagon or vehicle. At 1 h postdose, the mean plasma GRA1 concentration in these animals was 0.2 µM. *p<0.05; **p<0.01; and ***p<0.001 vs. glucagon-treated control animals.

In hGCGR mice given a single oral dose of 3 mg/kg GRA1, *in vivo* occupancy of hepatic hGCGR by ^125^I-glucagon was reduced by 64–73% for 1–8 h (Fig. 2A). A similar reduction in ^125^I-glucagon binding was observed in mice treated for 30 days with 3 mg/kg•day as a diet admixture (Fig. 2B). Administration of 15 µg/kg glucagon to hGCGR mice elicited a substantial increase in plasma glucose and this was significantly attenuated by pretreatment of animals with 3, 10, or 30 mg/kg GRA1 (Fig. 3A). The baseline-subtracted mean (±SEM) area-under-the-curve (0–50 min) for this glucagon-stimulated blood glucose excursion (AUC_0–50_) in hGCGR mice pretreated with vehicle was 1393±179 mg/dL•min. In mice pretreated with 3, 10 and 30 mg/kg GRA1, mean AUC_0–50_ was 812±122, 543±68 and 476±64 mg/dL•min, respectively. In mice that received sham injections of glucagon (containing only vehicle), mean AUC_0–50_ was 199±178 mg/dL•min.

In rhesus monkeys given a single oral dose of 2 mg/kg GRA1, 55% of the dose was absorbed, the apparent plasma terminal half-was 6.3 h, and as in mouse, nearly all absorbed drug was excreted unchanged via the bile. Treatment of rhesus monkeys with 1 mg/kg GRA1 reduced the magnitude of glucagon-stimulated blood glucose excursions by about 50%, an effect similar to that observed in hGCGR mice treated with 3 mg/kg (Fig. 3B).

### Effects of Chronic GRA1 Treatment in the hGCGR Diet-Induced Obese (DIO) Mouse

**Figure 4:**
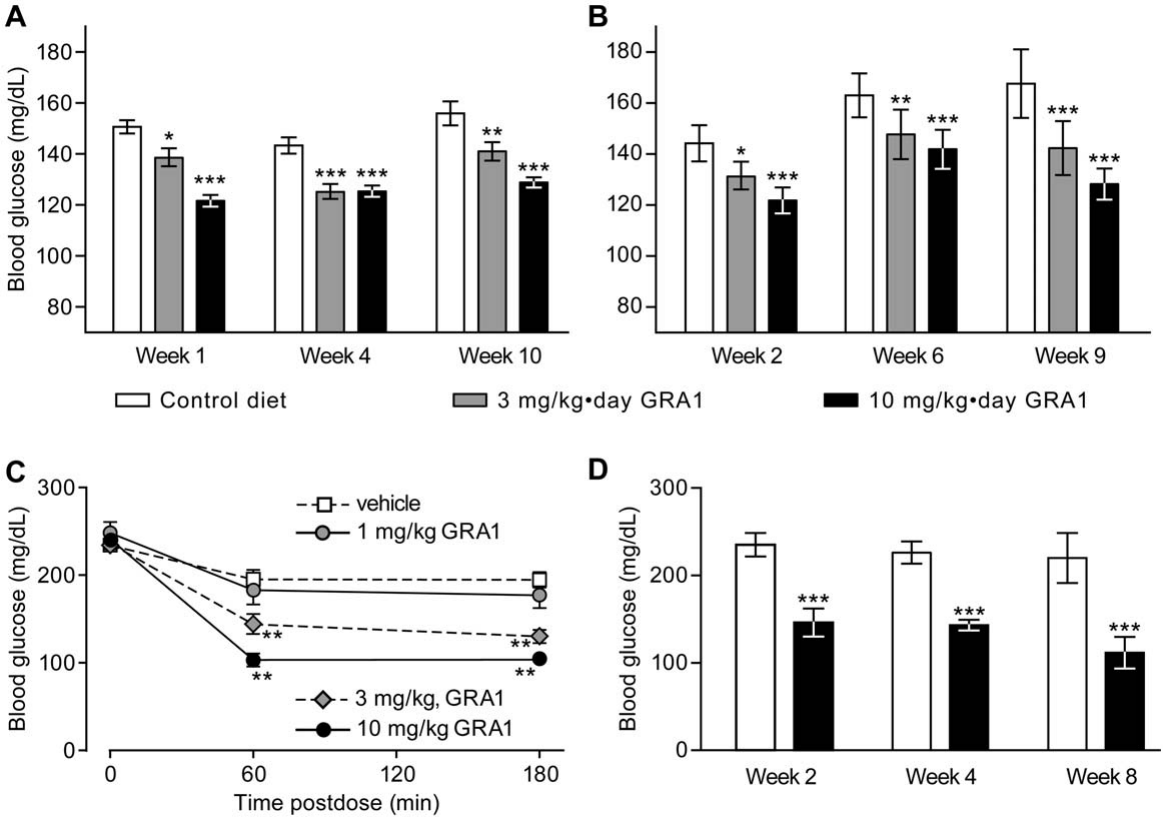
GRA1 lowers blood glucose in the DIO hGCGR and hGCGR.ob/ob mouse. Mean (±SEM) blood glucose concentrations in (A) non-fasted and (B) fasted DIO hGCGR mice treated with approximately 3 and 10 mg/kg·day GRA1 provided as a food admixture. (Additional data from this study are in Table 2). (C) Mean (±SEM) blood glucose concentrations in the hGCGR.ob/ob mouse following single oral doses of 1, 3, or 10 mg/kg GRA1 or vehicle. (D) Mean (±SEM) non-fasted blood glucose concentrations in hGCGR.ob/ob mice treated for 2–8 weeks with control diet or 10 mg/kg·day GRA1 administered as diet admixture. *p<0.05, **p<0.01, and ***p<0.001 in timepoint-matched comparisons with controls; n = 8 in each group.

Blood glucose levels are only moderately elevated in the hGCGR DIO mouse; in vehicle-treated animals, we generally observed blood glucose concentrations in the range of 140–170 mg/dL (Fig. 4A,B). Nonetheless, treatment of these mice for 1–10 weeks with 3 or 10 mg/kg•day GRA1 produced significant, sustained reductions in blood glucose. Plasma glucagon was increased by approximately 2-fold at either dose (Table 2). Food intake and body weight were not affected (data not shown). Plasma free fatty acids and triglycerides were reduced dose-dependently in hGCGR DIO mice treated with GRA1, but plasma cholesterol was unaffected.

**Table 2:** Plasma measurements in DIO hGCGR mice treated for 10 weeks with GRA1 administered as a food admixture.

		GRA1	
	Control diet	3 mg/kg•day	10 mg/kg•day
Insulin (ng/mL)	8.5±2.0	4.2±1.1	5.7±1.4
Glucagon (pg/mL)	164±24	374±53*	343±85
Triglyceride (mg/dL)	181±11	141±13*	122±10**
Free fatty acid (mM)	1.04±0.13	0.84±0.08	0.69±0.04*
Cholesterol (mg/dL)	233±15	255±17	213±8
LDL cholesterol (mg/dLl)	19.6±2.3	25.4±2.9	19.4±2.4
Non-LDL cholesterol (mg/dL)	205±12	216±16	186±7

All measurements were made in terminal plasma. Data are expressed as mean ± SEM; *p<0.05 and **p<0.01 in comparisons with the group on the control diet.

Immunohistochemical analysis of sections of pancreas prepared from treated and control animals found no significant differences in islet morphology or ratios of insulin-positive cells to glucagon-positive (data not shown).

### GRA1 Treatment Effects in the hGCGR.ob/ob Mouse

The hGCGR.*ob/ob* mouse had a diabetic phenotype comparable to that observed in the C57BL6/J-*ob/ob* mouse. They weighed approximately 50% more than their lean littermates and had significantly elevated levels of blood glucose, plasma insulin, and glucagon, relative to littermate controls (Table 3). Single doses of 3 and 10 mg/kg GRA1 lowered blood glucose significantly in these mice, compared with vehicle (Fig. 4C).

**Table 3:** The diabetic phenotype of the hGCGR.*ob/ob* mouse.

	Age (weeks)	hGCGR.*ob/ob*	littermate controls
Body weight (g)	4–5	32.0±0.7	21.9±0.5
Blood glucose, non-fasted (mg/dL)	7	203±17	110±4
Blood glucose, fasted (mg/dL)	7	97.1±4.0	71.3±3.5
Plasma insulin, non-fasted (ng/mL)	7	26.6±2.5	1.7±0.5
Plasma insulin, fasted (ng/mL)	7	13.3±1.4	0.78±1.2
Plasma glucagon, non-fasted (pg/mL)	7	192.3±17.0	53.3±11.4
Plasma glucagon, fasted (pg/mL)	7	109.0±13.5	53.3±16.4

The data are means ± SEM. In all comparisons, the difference between hGCGR.*ob/ob* mice and littermate controls was significant at p<0.001.

In hGCGR.*ob/ob* mice, mean (±SEM) plasma drug concentrations of GRA1 were 1.12±0.8 µM and 0.92±0.1 µM at 1 and 3 h, respectively, after administration of a single 3 mg/kg dose. In the chronic dosing experiment, similar plasma levels were achieved by administering 10 mg/kg•day GRA1 as a food admixture; after 5 weeks of chronic dosing, mean GRA1 plasma concentrations were 1.7±0.5 µM and 0.77±0.2 µM at 9AM and 4 PM, respectively.

The antihyperglycemic efficacy of GRA1 in hGCGR.*ob/ob* mice was significant and sustained. Assessed 2, 4, and 8 weeks after beginning treatment, non-fasted blood glucose levels remained 40–50% lower in GRA1-treated animals than in hGCGR.*ob/ob* mice fed the control diet (Fig. 4D). Fasting levels of blood glucose were similarly reduced by GRA1 treatment; at the end of 8 weeks of chronic treatment, mean (±SEM) fasted blood glucose was 189±36 mg/dL in hGCGR.*ob/ob* mice fed the control diet, versus 90.8±7.8 mg/dL in mice treated chronically with 10 mg/kg•day GRA1 (p<0.05).

Chronic treatment of hGCGR.*ob/ob* mice with GRA1 elicited moderate, but significant, increases in plasma glucagon and total GLP-1. At the end of 8 weeks of treatment, mean (±SEM) plasma glucagon was 1356±113 pg/mL in GRA1 treated animals compared with 959±90 pg/mL in animals fed the control diet (p<0.05). Total GLP-1 was 26.5±3.0 pM in the treated animals versus 15.6±2.0 pM in the controls (p<0.001). No differences were observed between these groups in food intake, body weight, or fasting insulin levels (data not shown).

### Effects of GRA1 Alone and Combined with Des-Fluoro-Sitagliptin in the Severely Diabetic hGCGR HFD/STZ Mouse

**Figure 5:**
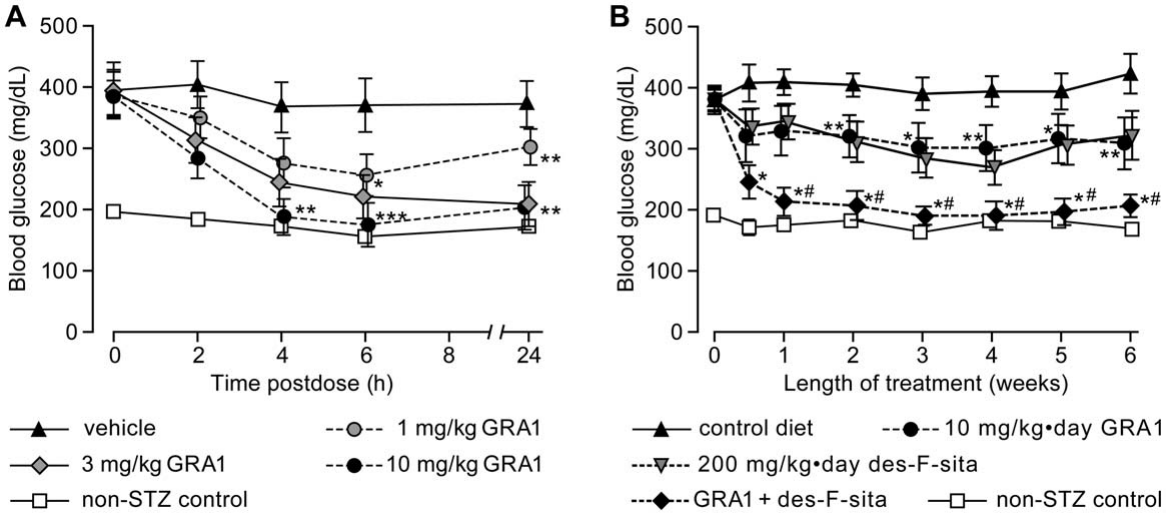
GRA1 lowers glucose in hGCGR HFD/STZ mice and further enhances the efficacy of a DPP-4 inhibitor. (A) Mean (±SEM) blood glucose hGCGR HFD/STZ mice treated with a single dose of 1, 3, or 10 mg/kg GRA1. (B) Non-fasted blood glucose concentrations in hGCGR HFD/STZ mice treated for 6 weeks with 10 mg/kg·day GRA1, 200 mg/kg·day des-fluoro-sitagliptin (des-F-sita), or the two agents in combination. (Additional data from this study are in Table 4.) *p<0.05, **p<0.01, and ***p<0.001 versus HFD/STZ controls; ^#^non-significant (p>0.05) versus non-STZ controls; n = 8–15 animals per group.

Untreated hGCGR HFD/STZ mice were severely diabetic, with blood glucose levels generally on the order of 400 mg/dL. Treatment of these mice with single doses of 3 and 10 mg/kg GRA1 reduced blood glucose significantly and substantially (Fig. 5A). Indeed, treatment with 10 mg/kg GRA1 resulted in blood glucose values that remained comparable to those observed in non-diabetic control animals for 24 h. During chronic dosing with GRA1, its antihyperglycemic efficacy in these mice was significant and sustained. Similar antihyperglycemic effect was observed in animals treated with 200 mg/kg·day des-fluoro-sitagliptin, and combination of these two agents resulted in additive lowering of blood glucose, to levels similar to those observed in non-diabetic control mice (Fig. 5B). Significant reductions in HbA1c and fasting blood glucose were also observed in this study and, more variably, modest effects on plasma triglycerides and free fatty acids (Table 4). Neither GRA1 nor des-fluoro-sitagliptin had a significant effect on body weight or food intake, either alone or in combination (data not shown).

**Table 4:** Plasma and tissue measurements from hGCGR HFD/STZ mice treated for 6 weeks with 10 mg/kg GRA1, des-fluoro-sitagliptin (des-F-sita), or GRA1 and des-fluoro-sitagliptin in combination.

	hGCGR HFD/STZ				hGCGR
	control diet	GRA1 (10 mg/kg)	Des-F-sita (200 mg/kg)	GRA1+Des-F-sita	control diet
6-h fasting blood glucose (mg/dL)^a^	323±33	297±39	283±34	161±23**	134±8**
HbA1c (%)^a^	6.1±0.4	5.6±0.4	5.0±0.4	4.1±0.2***	3.8±0.3**
Plasma glucagon (pg/ml)	133±21	209±24*	87±4	191±30	96±11
Active GLP-1 (pM)	2.5±0.1	2.7±0.1	2.9±0.2	7.0±1.1***	2.6±0.2
Inactive GLP-1 (pM)	8.1±1.1	8.6±0.4	3.2±0.2***	8.0±1.1	9.2±1.2
Triglyceride (mg/dL)	184±26	145±22*	161±15	109±10*	129±15*
Free fatty acid (mM)	1.4±0.2	1.3±0.3	1.1±0.1	0.7±0.1*	0.6±0.1**
Liver Triglyceride (µg/mg)	143±10	163±11	94±15*	108±9	93±23*

a Fasting blood glucose and HbA1c were measured after 5 weeks of treatment; all other measurements were made in terminal plasma and necropsy tissue. The data are means ± SEM; *p<0.05, **p<0.01 and ***p<0.001 for comparisons made with the hGCGR HFD/STZ group fed the control (drug-free) diet.

### GRA1 Treatment Altered Hepatic Expression of Genes Involved in Amino Acid Metabolism and Plasma Levels of Glucogenic Amino Acids

In monkeys treated for 1 day with 30 mg/kg GRA1 twice daily (n = 5 per group), there were approximately 1300 gene probe sets that met the criteria of showing a 1.2-fold change relative to vehicle animals and ANOVA-derived p value<0.05; after 6 days of treatment, 2100 probe sets met these criteria (data not shown). In addition to affecting hepatic expression of genes directly related to glucose metabolism (e.g. glucokinase), treatment with GRA1 elicited significant downregulation of 19 genes that are directly involved in amino acid metabolism (Table 5). Genes involved with amino acid metabolism were among the top enriched Gene Ontology Biological Process terms in the GRA1 induced liver signatures (data not shown).

**Table 5:** Genes involved in amino acid and glucose metabolism that were expressed differentially in rhesus monkey liver depending or whether animals received 30 mg/kg GRA1 or vehicle (yogurt without drug) twice daily for 6 days (n = 5 per group).

Gene Symbol	Gene Name	Day 1	p	Day 6	p
SDS	serine dehydratase	−8.8	**	−27.1	***
AASS	aminoadipate-semialdehyde synthase	−2.0	*	−3.0	**
OAT	ornithine aminotransferase	−1.9	*	−3.6	***
SLC7A2	solute carrier family 7 (cationic amino acid transporter, y+ system), member 2	−1.8	**	−2.4	***
TAT	tyrosine aminotransferase	−1.8	*	−1.7	ns
SLC1A2	solute carrier family 1 (glial high affinity glutamate transporter), member 2	−1.6	ns	−7.6	**
SDSL	serine dehydratase-like	−1.6	**	−1.5	ns
PAH	phenylalanine hydroxylase	−1.5	ns	−2.1	*
GPT	glutamic-pyruvate transaminase (alanine aminotransferase)	−1.5	***	−1.3	*
ASL	argininosuccinate lyase	−1.5	**	−1.6	**
GOT1	glutamic-oxaloacetic transaminase 1, soluble	−1.5	ns	−2.1	***
AGXT2L1	alanine-glyoxylate aminotransferase 2-like 1	−1.5	*	−1.8	**
BCKDHB	2-oxoisovalerate dehydrogenase subunit beta, mitochondrial-like	−1.4	**	−1.3	*
DDC	dopa decarboxylase (aromatic L-amino acid decarboxylase)	−1.4	*	−1.1	ns
GLS2	glutaminase 2 (liver, mitochondrial)	−1.3	ns	−1.6	*
KMO	kynurenine 3-monooxygenase (kynurenine 3-hydroxylase)	−1.3	*	−1.5	*
HPD	4-hydroxyphenylpyruvate dioxygenase	−1.3	*	−1.2	*
FH	fumarate hydratase	−1.2	**	−1.2	*
ARG1	arginase, liver	−1.1	ns	−1.3	**
GCKR	glucokinase (hexokinase 4) regulator	1.5	*	1.3	*
GCK	glucokinase (hexokinase 4)	2.4	ns	5.4	***

ns = not significant (p≥0.05).

The data are expressed as fold differences between treatment groups with negative values indicating reduced expression in animals treated with GRA1.

* p<0.05,

** p<0.01,

*** p<0.001.

In a follow-up experiment, adult rhesus monkeys (a different cohort; n = 8) were dosed with 30 mg/kg GRA1 once daily for 14 days and plasma samples were collected on days 0, 1, 7 and 14 for measurements of glucose, glucagon, and amino acid concentrations. Relative to vehicle-treated controls, plasma glucagon was increased by approximately 3-fold on both Day 1 and Day 7 of treatment, indicating rapid and sustained target engagement (Table 6). These normoglycemic animals were not rendered hypoglycemic by treatment with GRA1, although numerically lower values for mean fasting blood glucose were observed. Plasma insulin was unaffected (data not shown). Significant increases in plasma concentrations of several glucogenic amino acids were observed in the GRA1-treated animals on Day 1 and these increases were sustained through Day 6.

**Table 6:** Fasted plasma glucagon, glucose and amino acids in rhesus monkeys treated once daily with vehicle or 30 mg/kg GRA1.

	Day 0		Day 1		Day 7		Day 14	
	Vehicle	GRA1	Vehicle	GRA1	Vehicle	GRA1	Vehicle	GRA1
Glucagon (pg/ml)	284±56	381±74	294±151	921±16**	271±67	874±101***	323±136	984±186**
Glucose (mg/dL)	80.4±5.4	85.3±9.4	72.2±3.6	63.7±2.7	78.5±6.0	69.2±4.9	73.4±4.5	69.2±3.8
Aspartic acid (µM)	5.7±1.1	5.3±1.3	5.4±0.7	5.6±0.7	5.8±1.0	6.7±1.2	4.3±0.5	5.9±0.8
Hydroxyproline (µM)	12.1±1.9	12.9±2.3	11.1±1.6	15.4±2.3	12.0±2.0	23.3±5.3	13.0±2.3	15.3±2.3
Glutamic acid (µM)	47.7±7.7	48.1±3.6	46.4±7.1	71.9±5.7*	53.6±8.6	98.9±15.2*	63.0±8.5	91.9±10.0*
Asparagine (µM)	42.5±9.4	61.2±17.8	36.6±9.6	42.5±8.3	52.5±18.9	54.2±10.8	26.3±4.9	33.8±4.8
Serine (µM)	122±7	123±8	143±6	228±24**	125±8	256±41*	144±10	217±28*
Histidine (µM)	112±8	121±11	121±7	153±14*	118±9	170±15**	132±8	187±21*
Glycine (µM)	304±21	300±32	360±17	533±70*	303±28	558±74**	356±38	531±76
Glutamine (µM)	575±53	597±66	647±57	986±150	639±58	1186±164**	736±55	1151±191
Arginine (µM)	115±11	124±15	136±12	237±33*	123±13	341±76*	145±11	282±54*
Threonine (µM)	95.8±6.9	95.2±5.9	116.2±7.1	179.1±25.1*	92.8±6.7	160.9±21.3*	109.8±10.9	155.3±23.3
Alanine (µM)	195±28	200±21	209±23	500±79**	184±21	456±68**	241±30	361±51
Proline (µM)	111±18	118±13	144±19	326±54**	122±8	490±95**	181±21	415±84*
Tyrosine (µM)	72.8±5.1	77.2±6.8	86.0±2.8	161.4±26.8*	97.3±14.3	110.6±7.9	105.5±17.3	105.8±9.9
Valine (µM)	289±24	256±21	251±15	252±33	279±43	260±17	293±30	276±20
Isoleucine (µM)	102.8±10.6	98.2±8.3	90.3±8.9	72.4±7.9	96.7±17.5	88.6±8.2	95.7±17.7	89.6±7.8
Leucine (µM)	148±15	147±9	136±10	164±24	170±32	215±44	132±19	218±36*
Lysine (µM)	193±22	191±25	227±24	390±56*	195±24	340±71	215±21	375±70*
Phenylalanine (µM)	49.0±2.0	47.8±2.4	50.3±2.2	52.6±7.0	49.5±2.3	49.7±3.3	48.2±2.7	51.5±1.8
Methionine (µM)	22.5±1.9	22.9±2.7	25.8±2.0	50.2±10.0*	23.5±1.6	36.1±5.6*	24.1±3.1	34.7±6.3

Data expressed as mean ± SEM; *p<0.05, **p<0.01 and ***p<0.001 vs. vehicle-treated animals at indicated days.

## Discussion

Relative or absolute insulin deficiency is a well-known and important contributor to the pathophysiology of diabetes. Dysregulation of glucagon has received less attention in this regard, but is also thought to play an important role in diabetes [3], [6]. Hepatic overproduction of glucose is a prominent contributor to hyperglycemia in patients with type 2 diabetes, and this overproduction may be a consequence of chronic and postprandial hyperglucagonemia [33], [34]. Suppression of glucagon action on target organs may be a secondary mechanism underlying the antihyperglycemic efficacy of GLP-1 analogs and DPP-4 inhibitors [31], but there are no approved treatments for diabetes that directly target glucagon secretion or glucagon action in target organs.

A number of approaches directed towards blocking glucagon receptor activity, including small molecule antagonists, are currently in development as antihyperglycemic agents [8], [9], [35], [36]. The present data indicate that GRA1 is a potent and selective GRA that has strong antihyperglycemic efficacy in animal models of diabetes and favorable pharmacokinetic characteristics.

In this study, we used a mouse model in which the murine GCGR gene has been replaced by the hGCGR ortholog. We have previously used this model to demonstrate the ability of other GRAs to block the hyperglycemic effects of exogenously administered glucagon [17], [20], [21], [37]–[39]. The present data show that that GRA1 has this glucagon-antagonizing action in the hGCGR mouse and also in the rhesus monkey, a species in which the *in vitro* anti-GCGR potency of GRA1 is much closer to that observed with the hGCGR. We further observed that GRA1 produced significant glucose reduction acutely and chronically in DIO hGCGR mice, even though these mice were only moderately hyperglycemic. To evaluate GRA1 efficacy in models with more elevated blood glucose, we first tested it in hGCGR.*ob/ob* mice, a line combining features like hyperinsulinemia, hyperglucagonemia and mild hyperglycemia [28]. Glucose levels in hGCGR.*ob/ob* mice are comparable to those observed in many patients with mild to moderate or poorly controlled type 2 diabetes. In addition, we also generated HFD/STZ hGCGR mice which have compromised β-cell function and more severe hyperglycemia. In both models, GRA1 showed substantial and durable glucose lowering. Furthermore, studies in the HFD/STZ model demonstrated the feasibility of combining GRA1 with a DPP-4 inhibitor to achieve additional glycemic benefit, which is likely due to DPP-4 inhibitor-mediated stabilization of GLP-1, the secretion of which is enhanced due to GCGR antagonism [20].

Similar to what was observed in early GRA studies [20], chronic GRA1 treatments led to only moderate increases of plasma glucagon, GLP-1 and was devoid of the α-cell hyperplasia that is prominent in GCGR knockout mice and animals treated with GCGR antisense oligonucleotide [13], [14]. The mice exposed to GRA1 chronically in this study did not show elevations of liver triglycerides, plasma lipids (Tables 2 and 4) or liver enzymes such as aspartate aminotransferase (data not shown). In addition, no hypoglycemia was observed in these studies even when GRA1 was administered chronically to normoglycemic hGCGR mice, normoglycemic monkeys, and hGCGR DIO mice that were only moderately hyperglycemic. This observation should be interpreted with caution, however, because antagonism of the GCGR could hypothetically lead to hypoglycemia in situations in which glucagon was contributing importantly to a counterregulatory response to excessive insulin or an insulin secretagogue [40]. Under the conditions of the present experiments, however, treatment with GRA1 did not elicit hypoglycemia.

A close GRA analog was recently evaluated in clinical studies and its antihyperglycemic efficacy confirmed in humans (unpublished data). In these same studies, however, some untoward effects on blood lipids were observed, effects that had not been seen in preclinical species [41]–[43]. It was also observed that combination of this GRA (MK-0893) with the DPP-4 inhibitor sitagliptin did not result in significant additional glucose-lowering efficacy. While it cannot be ruled out that these differences may have arisen because different compounds were studied, it is more likely that some important differences exist between rodent and human in GCGR-mediated signal transduction and downstream metabolic events. Further studies are on-going to provide a better understanding of these preclinical/clinical differences.

In the present study, gene-expression profiling revealed that GRA1 treatment has prominent effects on amino acid metabolism, in addition to its effect on genes that are directly involved in glucose metabolism. Although the underlying mechanisms are not well understood, glucagon is known to play an important role in amino acid metabolism, including stimulation of gluconeogenesis from amino acid substrates [23]–[25]. Hypoaminoacidemia, weight loss, and muscle wasting are key features of glucagonoma in humans [44]; conversely, lean body mass and circulating amino acids are increased in mice with targeted deletion of the GCGR gene [13], [22], [24], [25], [26]. The present observation of down-regulation of genes related to amino acid catabolism is therefore highly consistent with GRA1's intended mechanism of action. The present data provide evidence that these effects are a direct effect consequence of blocking glucagon action, and not a developmental consequence of gene deletion, and the present data further indicate that this relationship between glucagon and amino acid metabolism is conserved across species.

In summary, GRA1 showed a robust glucose efficacy and durability in animal models that have different levels of glucose and metabolic characteristics. In addition, by antagonizing glucagon-mediated amino acid catabolism under hyperglucagonemia, such as what is seen in some poorly controlled diabetic patients [45], GRA1 could help to alleviate conditions such as hypoaminoacidemia and muscle wasting [23].

## References

[1] Unger RH, Aguilar-Parada E, Muller WA, Eisentraut AM (1970) Studies of pancreatic alpha cell function in normal and diabetic subjects. J Clin Invest 49: 837–848. 4986215 10.1172/JCI106297 PMC322540

[2] Burcelin R, Katz EB, Charron MJ (1996) Molecular and cellular aspects of the glucagon receptor: role in diabetes and metabolism. Diabetes Metab 22: 373–396. 8985646

[3] Jiang G, Zhang BB (2003) Glucagon and regulation of glucose metabolism. Am J Physiol Endocrinol Metab 284: E671–678. 12626323 10.1152/ajpendo.00492.2002

[4] Unger RH (1978) Role of glucagon in the pathogenesis of diabetes: the status of the controversy. Metabolism 27: 1691–1709. 360007 10.1016/0026-0495(78)90291-3

[5] Toft I, Gerich JE, Jenssen T (2002) Autoregulation of endogenous glucose production during hyperglucagonemia. Metabolism 51: 1128–1134. 12200756 10.1053/meta.2002.34702

[6] Unger RH, Cherrington AD (2012) Glucagonocentric restructuring of diabetes: a pathophysiologic and therapeutic makeover. J Clin Invest 122: 4–12. 22214853 10.1172/JCI60016 PMC3248306

[7] Zhang BB, Moller DE (2000) New approaches in the treatment of type 2 diabetes. Curr Opin Chem Biol 4: v461–467. 10.1016/s1367-5931(00)00103-4 10959776

[8] Sloop KW, Michael MD, Moyers JS (2005) Glucagon as a target for the treatment of Type 2 diabetes. Expert Opin Ther Targets 9: 593–600. 15948676 10.1517/14728222.9.3.593

[9] Bagger JI, Knop FK, Holst JJ, Vilsboll T (2011) Glucagon antagonism as a potential therapeutic target in type 2 diabetes. Diabetes Obes Metab 13: 965–971. 21615669 10.1111/j.1463-1326.2011.01427.x

[10] Lok S, Kuijper JL, Jelinek LJ, Kramer JM, Whitmore TE, et al (1994) The human glucagon receptor encoding gene: structure, cDNA sequence and chromosomal localization. Gene 140: 203–209. 8144028 10.1016/0378-1119(94)90545-2

[11] Parker JC, Andrews KM, Allen MR, Stock JL, McNeish JD (2002) Glycemic control in mice with targeted disruption of the glucagon receptor gene. Biochem Biophys Res Commun 290: 839–843. 11785978 10.1006/bbrc.2001.6265

[12] Gelling RW, Du XQ, Dichmann DS, Romer J, Huang H, et al (2003) Lower blood glucose, hyperglucagonemia, and pancreatic alpha cell hyperplasia in glucagon receptor knockout mice. Proc Natl Acad Sci U S A 100: 1438–1443. 12552113 10.1073/pnas.0237106100 PMC298791

[13] Liang Y, Osborne MC, Monia BP, Bhanot S, Gaarde WA, et al (2004) Reduction in glucagon receptor expression by an antisense oligonucleotide ameliorates diabetic syndrome in db/db mice. Diabetes 53: 410–417. 14747292 10.2337/diabetes.53.2.410

[14] Sloop KW, Cao JX, Siesky AM, Zhang HY, Bodenmiller DM, et al (2004) Hepatic and glucagon-like peptide-1-mediated reversal of diabetes by glucagon receptor antisense oligonucleotide inhibitors. J Clin Invest 113: 1571–1581. 15173883 10.1172/JCI20911 PMC419489

[15] Conarello SL, Jiang G, Mu J, Li Z, Woods J, et al (2007) Glucagon receptor knockout mice are resistant to diet-induced obesity and streptozotocin-mediated beta cell loss and hyperglycaemia. Diabetologia 50: 142–150. 17131145 10.1007/s00125-006-0481-3

[16] Petersen KF, Sullivan JT (2001) Effects of a novel glucagon receptor antagonist (Bay 27-9955) on glucagon-stimulated glucose production in humans. Diabetologia 44: 2018–2024. 11719833 10.1007/s001250100006

[17] Qureshi SA, Rios Candelore M, Xie D, Yang X, Tota LM, et al (2004) A novel glucagon receptor antagonist inhibits glucagon-mediated biological effects. Diabetes 53: 3267–3273. 15561959 10.2337/diabetes.53.12.3267

[18] Rivera N, Everett-Grueter CA, Edgerton DS, Rodewald T, Neal DW, et al (2007) A novel glucagon receptor antagonist, NNC 25-0926, blunts hepatic glucose production in the conscious dog. J Pharmacol Exp Ther 321: 743–752. 17308040 10.1124/jpet.106.115717

[19] Winzell MS, Brand CL, Wierup N, Sidelmann UG, Sundler F, et al (2007) Glucagon receptor antagonism improves islet function in mice with insulin resistance induced by a high-fat diet. Diabetologia 50: 1453–1462. 17479245 10.1007/s00125-007-0675-3

[20] Mu J, Jiang G, Brady E, Dallas-Yang Q, Liu F, et al (2011) Chronic treatment with a glucagon receptor antagonist lowers glucose and moderately raises circulating glucagon and glucagon-like peptide 1 without severe alpha cell hypertrophy in diet-induced obese mice. Diabetologia 54: 2381–2391. 21695571 10.1007/s00125-011-2217-2

[21] Shen DM, Brady EJ, Candelore MR, Dallas-Yang Q, Ding VD, et al (2011) Discovery of novel, potent, selective, and orally active human glucagon receptor antagonists containing a pyrazole core. Bioorg Med Chem Lett 21: 76–81. 21147532 10.1016/j.bmcl.2010.11.074

[22] Flakoll PJ, Borel MJ, Wentzel LS, Williams PE, Lacy DB, et al (1994) The role of glucagon in the control of protein and amino acid metabolism in vivo. Metabolism 43: 1509–1516. 7990704 10.1016/0026-0495(94)90009-4

[23] Charlton MR, Adey DB, Nair KS (1996) Evidence for a catabolic role of glucagon during an amino acid load. J Clin Invest 98: 90–99. 8690809 10.1172/JCI118782 PMC507404

[24] Yang J, MacDougall ML, McDowell MT, Xi L, Wei R, et al (2011) Polyomic profiling reveals significant hepatic metabolic alterations in glucagon-receptor (GCGR) knockout mice: implications on anti-glucagon therapies for diabetes. BMC Genomics 12: 281. 21631939 10.1186/1471-2164-12-281 PMC3130710

[25] Lee Y, Wang MY, Du XQ, Charron MJ, Unger RH (2011) Glucagon receptor knockout prevents insulin-deficient type 1 diabetes in mice. Diabetes 60: 391–397. 21270251 10.2337/db10-0426 PMC3028337

[26] Xiong Y, Guo J, Candelore MR, Liang R, Miller C, et al (2012) Discovery of a novel glucagon receptor antagonist N-[(4-{(1S)-1-[3-(3, 5-Dichlorophenyl)-5-(6-methoxynaphthalen-2-yl)-1H-pyrazol-1-yl]ethyl}phenyl)carbo nyl]-beta-alanine (MK-0893) for the treatment of type II diabetes. J Med Chem doi:10.1021/jm300579z 10.1021/jm300579z 22708876

[27] Shiao LL, Cascieri MA, Trumbauer M, Chen H, Sullivan KA (1999) Generation of mice expressing the human glucagon receptor with a direct replacement vector. Transgenic Res 8: 295–302. 10621976 10.1023/a:1008922521461

[28] Coleman DL, Hummel KP (1973) The influence of genetic background on the expression of the obese (Ob) gene in the mouse. Diabetologia 9: 287–293. 4588246 10.1007/BF01221856

[29] Dallas-Yang Q, Shen X, Strowski M, Brady E, Saperstein R, et al (2004) Hepatic glucagon receptor binding and glucose-lowering in vivo by peptidyl and non-peptidyl glucagon receptor antagonists. Eur J Pharmacol 501: 225–234. 15464082 10.1016/j.ejphar.2004.08.023

[30] Cohen SM, Duffy JL, Miller C, Kirk BA, Candelore MR, et al (2006) Direct observation (NMR) of the efficacy of glucagon receptor antagonists in murine liver expressing the human glucagon receptor. Bioorg Med Chem 14: 1506–1517. 16256355 10.1016/j.bmc.2005.10.008

[31] Mu J, Woods J, Zhou YP, Roy RS, Li Z, et al (2006) Chronic inhibition of dipeptidyl peptidase-4 with a sitagliptin analog preserves pancreatic beta-cell mass and function in a rodent model of type 2 diabetes. Diabetes 55: 1695–1704. 16731832 10.2337/db05-1602

[32] Zhao W, Fong O, Muise ES, Thompson JR, Weingarth D, et al (2010) Genome-wide expression profiling revealed peripheral effects of cannabinoid receptor 1 inverse agonists in improving insulin sensitivity and metabolic parameters. Mol Pharmacol 78 3: 350–359. 20530130 10.1124/mol.110.064980

[33] Reaven GM, Chen YD, Golay A, Swislocki AL, Jaspan JB (1987) Documentation of hyperglucagonemia throughout the day in nonobese and obese patients with noninsulin-dependent diabetes mellitus. J Clin Endocrinol Metab 64: 106–110. 3536980 10.1210/jcem-64-1-106

[34] Shah P, Vella A, Basu A, Basu R, Schwenk WF, et al (2000) Lack of suppression of glucagon contributes to postprandial hyperglycemia in subjects with type 2 diabetes mellitus. J Clin Endocrinol Metab 85: 4053–4059. 11095432 10.1210/jcem.85.11.6993

[35] Yan H, Gu W, Yang J, Bi V, Shen Y, et al (2009) Fully human monoclonal antibodies antagonizing the glucagon receptor improve glucose homeostasis in mice and monkeys. J Pharmacol Exp Ther 329: 102–111. 19129372 10.1124/jpet.108.147009

[36] Shen DM, Lin S, Parmee ER (2011) A survey of small molecule glucagon receptor antagonists from recent patents (2006–2010). Expert Opin Ther Pat 21: 1211–1240. 21635155 10.1517/13543776.2011.587001

[37] Duffy JL, Kirk BA, Konteatis Z, Campbell EL, Liang R, et al (2005) Discovery and investigation of a novel class of thiophene-derived antagonists of the human glucagon receptor. Bioorg Med Chem Lett 15: 1401–1405. 15713396 10.1016/j.bmcl.2005.01.003

[38] Shen DM, Zhang F, Brady EJ, Candelore MR, Dallas-Yang Q, et al (2005) Discovery of novel, potent, and orally active spiro-urea human glucagon receptor antagonists. Bioorg Med Chem Lett 15: 4564–4569. 16102966 10.1016/j.bmcl.2005.06.101

[39] Kim RM, Chang J, Lins AR, Brady E, Candelore MR, et al (2008) Discovery of potent, orally active benzimidazole glucagon receptor antagonists. Bioorg Med Chem Lett 18: 3701–3705. 18539028 10.1016/j.bmcl.2008.05.072

[40] Cryer PE (2008) Hypoglycemia: still the limiting factor in the glycemic management of diabetes. Endocr Pract 14: 750–756. 18996798 10.4158/EP.14.6.750

[41] Engel SS, Xu L, Andryuk PJ, Davies MJ, Amatruda J, et al (2011) Efficacy and tolerability of MK-0893, a glucagon receptor antagonist (GRA), in patients with type 2 diabetes (T2DM). Diabetes 60: A85.

[42] Ruddy M, Pramanik B, Lunceford SL, Cilissen C, Stoch A, et al (2011) Inhibition of glucagon-induced hyperglycemia predicts glucose lowering efficacy of a glucagon receptor antagonist, MK-0893, in type 2 diabetes (T2DM). Diabetes 60: A85.

[43] Engel SS, Teng R, Edwards RJ, Davies MJ, Kaufman KD, et al. (2011) Efficacy and safety of the glucagon receptor antagonist, MK-0893, in combination with metformin or sitagliptin in patients with type 2 diabetes mellitus 47th EASD Abstract 191.

[44] Eldor R, Glaser B, Fraenkel M, Doviner V, Salmon A, et al (2011) Glucagonoma and the glucagonoma syndrome - cumulative experience with an elusive endocrine tumour. Clin Endocrinol (Oxf) 74: 593–598. 21470282 10.1111/j.1365-2265.2011.03967.x

[45] Hebert SL, Nair KS (2010) Protein and energy metabolism in type 1 diabetes. Clin Nutr 29: 13–17. 19788950 10.1016/j.clnu.2009.09.001 PMC2822109

